# Animal models of autism spectrum disorder: Insights into genetic, structural and environmental models

**DOI:** 10.17221/87/2024-VETMED

**Published:** 2025-07-25

**Authors:** Dariya Chivchibashi-Pavlova, Kameliya Bratoeva

**Affiliations:** Department of Physiology and Pathophysiology, Faculty of Medicine, Medical University – Varna, Varna, Bulgaria

**Keywords:** animal experimental models, novel experimental models, primate models, rodent models, zebrafish models

## Abstract

Autism spectrum disorder (ASD) is a group of human neurodevelopmental disorders with significant global prevalence. Deficits in social communication and interaction and repetitive, stereotyped patterns of behaviour characterise ASD. The aetiology of ASD is unclear, but several genetic and environmental risk factors, either alone or in combination, are implicated in its development. To date, the underlying pathogenic mechanisms of ASD remain incompletely understood due to its heterogeneity. To better understand the pathogenesis of ASD, various animal models have been developed. The use of animals in ASD research allows the exploration of the biological substrates of social behaviour, cognition, and reward sensitivity, which are key components of ASD symptoms. This review outlines the commonly employed animal models in ASD research and explores their applications and the associated challenges.

## INTRODUCTION

At present, “autism spectrum disorders” (ASD) is an umbrella term used to describe a clinically heterogeneous group of human neurodevelopmental disorders characterised by deficits in social interaction, verbal and non-verbal communication difficulties, restrictive and repetitive stereotypic behavioural patterns, and narrow interests (Patel et al. 2018; Li et al. 2021; Sauer et al. 2021). ASD is one of the most disabling developmental disorders (Talantseva et al. 2023) with significant global prevalence. It affects approximately 0.6–1% of the worldwide population (Salari et al. 2022; Andersen-Civil et al. 2023). Nevertheless, practical methods for diagnosing and treating ASD remain insufficient, primarily because of the significant heterogeneity of the disorder. ASD presents not only a medical challenge for individuals but also a pressing social concern, placing substantial mental and financial strain on families and society as a whole (Manoli and State 2021). Thus, comprehensive studies on the pathophysiology of ASD are essential for providing theoretical and experimental foundations to advance new clinical diagnostic, treatment, and intervention strategies (Li et al. 2021).

Considering the challenges in obtaining samples from ASD patients, experimental models that replicate the clinical features of ASD are the best choice for exploring the pathogenesis of the disorder (Erdogan et al. 2017; Wintler et al. 2020; Li et al. 2021). To date, numerous animal models for ASD have been developed, each possessing distinct advantages and disadvantages. In this review, we discuss the applications of standard ASD animal models. The latter can be divided into three major groups: 1) Genetic animal models associated with the study of the disease in genetically modified animals; 2) Structural injury-induced animal models based on the destruction of areas in the central nervous system; and 3) Environmental-induced animal models – animal models in which chemical influences or biological manoeuvre are applied during early development (ontogenesis) (Lavrov and Shabanov 2018; Li et al. 2021; Ornoy et al. 2024).

## GENETIC ANIMAL MODELS FOR ASD

Genetic research has identified numerous genes associated with ASD, with over 900 genes linked to the disorder (Miles 2011; Pensado-Lopez et al. 2020). Understanding the pathogenesis of ASD involves detecting copy number variations and point mutations, as well as identifying rare variants in synaptic cell adhesion proteins and their associated pathways. These genetic pathways can be experimentally modelled. However, the complex genetic framework of ASD, including genomic abnormalities, *de* *novo* mutations, and prevalent genetic variants, makes translating genetic risk into biological mechanisms a challenge (Erdogan et al. 2017).

Rodent models have historically been the preferred choice for studying the genetic alterations present in neurodevelopmental and neurodegenerative human diseases, including ASD. Additionally, multiple mouse strains exhibiting ASD-like behaviours were used to identify factors behind ASD, making mice also the preferred model. Subsequently, the development of rat knockout models marked a significant advancement (Ornoy et al. 2024). Initial knockout rat models included those associated with ASD resulting from both genetic syndromes caused by mutations in a single gene and nonsyndromic mutations. Examples of syndromic models are those associated with a mutation in the fragile X messenger ribonucleoprotein 1, *FMR1* gene, and the methyl CpG binding protein 2, *MECP2 *gene, as well as nonsyndromic models linked to mutations in the neuroligin 3 (*NLGN3*) and neurexin 1 (*NRXN1*) genes (Pietri et al. 2013; Schepici et al. 2019; Pensado-Lopez et al. 2020; Zhu et al. 2023). Although rodent models have been commonly used to study human genetic disorders, significant evolutionary differences, such as brain anatomy, cognitive abilities, and behaviour, raise questions about their translational potential (Zhao et al. 2018). Currently, apart from rodent genetic models, zebrafish and nonhuman primates, such as cynomolgus monkey models, are emerging as a significant supplementary model in translational neuroscience (Pietri et al. 2013; Liu et al. 2016; Schepici et al. 2019; Kedra et al. 2020; Pensado-Lopez et al. 2020; Li et al. 2021; Zhu et al. 2023). Transgenic monkey models offer improved face and constant validity for evaluating ASD-like phenotypes, as they are more closely related to humans than rodent or zebrafish models (Li et al. 2021). However, cost, the slow reproductive cycle of macaques, and limited phenotype tools hinder the use of these animals in experiments. Additionally, ethical concerns and non-genetic factors, such as diet, environment, and socioeconomic status, further complicate research outcomes (Zhao et al. 2018).

Among genetic syndromes associated with ASD, the more prevalent conditions include fragile X syndrome, caused by a mutation in the *FMR1* gene (Miles 2011; Pensado-Lopez et al. 2020); Rett syndrome, linked to mutations in the *MECP2* gene; Tuberous sclerosis, associated with mutations in *TSC1* or *TSC2*; and Timothy syndrome, which arises from a mutation in the *CACNA1C* gene (Ergaz et al. 2016). Fragile X syndrome is considered the most prevalent inherited form of ASD (Miles 2011; Pensado-Lopez et al. 2020). Although these syndromes are primarily recognised as human diseases, they are not exclusive to humans in terms of research models. The majority of these models exhibit an ASD phenotype that closely mirrors the clinical features observed in human patients with ASD (Li et al. 2021; Ornoy et al. 2024).

Examples of animal models of syndromic disorders predisposing to ASD among humans are summarised in Table 1.

**Table 1:** Examples of animal models of syndromic disorders predisposing to ASD among humans

Human syndrome	Gene mutation (human)	Animal model	Behavioural ASD features modelled	Study
Fragile X syndrome	*FMR1*	mice, *Fmr1* –/–	impaired social communication, repetitive and restricted behaviours, reduced anxiety levels, hyperactivity, and seizures	Zang et al. (2009); Oddi et al. (2015)
Fragile X syndrome	*FMR1*	rats, *Fmr1* –/–	impaired social communication, repetitive and restricted behaviours, and intact fear responses, along with normal sensorimotor gating	Hamilton et al. (2014)
Fragile X syndrome	*FMR1*	zebrafish, *Fmr1* –/–	anxiety-like behaviours, hyperactivity, and heightened sensitivity to auditory and visual stimuli	Zhu et al. (2023)
Rett syndrome	*MECP2*	mice, *Mecp2 +/−*	decreased anxiety, narrow interests, diminished pain sensitivity, and normal olfaction.	Samaco et al. (2013)
Rett syndrome	*MECP2*	mice, *Mecp2 *methylation	impaired social communication, increased grooming, enhanced anxiety and/or depression, and poor performance in memory tasks	Lu et al. (2020)
Rett syndrome	*MECP2*	rats, *Mecp2* –/–	defects in social interaction, poor memory, and task performance	Wu et al. (2016)
Rett syndrome	*MECP2*	zebrafish, *mecp2* –/–	behavioural impairments	Pietri et al. (2013)
Rett syndrome	*MECP2*	cynomolgus monkeys, *MeCP2*	repetitive circular locomotion, increased stress responses, diminished social engagement, and mild improvements in cognitive abilities	Liu et al. (2016)
Tuberous sclerosis	*TSC1, TSC2*	mice, *Tsc1 +/–* and *Tsc2 +/–*	impaired social interactions	Sato et al. (2012)
Tuberous sclerosis	*TSC1, TSC2*	zebrafish, *tsc2 ^vu242/vu242^ *mutants	seizures, anxiety-like behaviour	Kedra et al. (2020)
Timothy syndrome	*CACNA1C*	mice, *Ts2*, G406 mutation in the CaV1.2L-ty	impaired social communication, repetitive and restricted behaviours, increased fear	Bader et al. (2011)
Timothy syndrome	*CACNA1C*	mice, G406R mutation in L-type Ca^2+^ channels	behavioural impairments	Horigane et al.(2020)

ASD = autism spectrum disorder

Thus, *Fmr1-*mutant mice show difficulties with social interactions, repetitive and stereotypic behaviours, and reduced anxiety, hyperactivity, and seizures (Zang et al. 2009; Oddi et al. 2015). In *Fmr1-*knockout rat models, impaired social interactions and repetitive behaviours were also observed, but there was no effect on fear responses or sensorimotor gating (Hamilton et al. 2014). In zebrafish, mutations in the *fmr1* result in anxiety-like behaviours, hyperactivity, and heightened sensitivity to auditory and visual stimuli (Zhu et al. 2023).

Mouse models with *Mecp2 *mutations replicate the key symptoms of Rett syndrome observed in humans (Li et al. 2021). According to multiple research studies, *Mecp2-*knockout mice tend to develop normally for a month, after which they show hypoactivity, seizures, repetitive movements, and social deficits (Samaco et al. 2013; Erdogan et al. 2017; Lu et al. 2020; Li et al. 2021). Rats affected by the *Mecp2* mutation also show ASD-like impairments, such as defects in social interaction and poor performance in memory tasks (Wu et al. 2016). Interestingly, non-traditional ASD models such as zebrafish and monkeys with the *mecp2/MeCp2* gene mutation also show ASD-like behavioural impairments (Pietri et al. 2013; Li et al. 2021). Liu et al. (2016) reported that *MeCP2* transgenic cynomolgus monkeys over-expressing human *MECP2* in the brain display an increased tendency for repetitive circular movements, heightened stress responses, diminished social engagement, and mild impairments of cognitive abilities.

In mouse models with *Tsc1* or *Tsc2 *mutations, animals display ASD-like behaviours, such as impaired social interactions and altered vocalisations (Sato et al. 2012; Li et al. 2021), while zebrafish models exhibit seizures and anxiety-like behaviours (Kedra et al. 2020).

Many mouse models have been developed with a high degree of face validity for the behavioural signs associated with Timothy syndrome. Timothy syndrome mouse mutants show ASD-like features such as impaired social interactions (Bader et al. 2011; Horigane et al. 2020), repetitive and stereotypic behaviours, and increased fear (Bader et al. 2011).

Additionally, many ASD-associated genes regulate synaptic adhesion, disrupting the balance between excitatory and inhibitory control in neural pathways. Synaptic cell adhesion molecules such as neurexins, neuroligins, and contactins are essential for synapse formation and function. Neuroligins (NLGNs), a large group of transmembrane proteins, are found on the postsynaptic membrane of glutamatergic or GABAergic synapses. The *NLGN* gene family comprises five distinct human genes, with neuroligin 4 X-linked *(NLGN4X)* and *NLGN3* mutations linked to ASD (Onay et al. 2017). These mutations produce altered proteins that reduce cell surface binding to Neurexin (NRXN), forming the basis for animal models. Neurexins, primarily presynaptic transmembrane proteins, are encoded by three genes (*NRXN1*, *NRXN2*, and *NRXN3*) and form complexes with neuroligins. According to studies by Ishizuka and colleagues (Ishizuka et al. 2020) and Onay and colleagues, *NRXN1* gene mutations may be pathogenetically associated with ASD (Onay et al. 2017). Glycosylphosphatidylinositol-anchored immunoglobulin proteins like Contactin 4 are involved in myelination, synapse formation, and plasticity, with disruptions linked to ASD. Genes for scaffolding proteins such as *SHANK* (*SHANK1-3*) are also crucial for synaptic function; *SHANK3* mutations have been linked to ASD, resulting in specific behavioural phenotypes in mutant mice (Erdogan et al. 2017). Studies on *Shank3*-deficient mice suggest that *Shank3* deficiency may lead to abnormalities in gamma-aminobutyric acidergic neurons (GABAergic neurons), impairing the GABAergic neurotransmission, which is pathogenetically linked to ASD (Bacova et al. 2025).

## ANIMAL MODELS OF STRUCTURAL BRAIN INJURY IN ASD

Similar to autistic individuals, animals with alterations in specific brain regions exhibit ASD-like behaviour (Erdogan et al. 2017), as these alterations can affect various areas involved in speech production, comprehension, and sensory processing, thereby contributing to the diverse clinical features observed in ASD (Khadem-Reza and Zare 2022). Decades of research have identified key hallmarks of ASD and crucial brain regions involved, particularly those in the “social brain”, including the prefrontal cortex, amygdala, hippocampus, limbic system, and dopaminergic pathways. Additionally, increasing evidence also points to the cerebellum’s role in cognitive and social functions, highlighting its involvement in ASD, though the extent of this role is still unclear (Mapelli et al. 2022).

Traditional anatomical lesion models have helped identify brain regions involved in neurological disorders. However, such models fail to accurately replicate ASD due to the complexity of human development and the diversity of ASD phenotypes, which likely involve multiple neural circuits and brain regions (Kim et al. 2016).

## ANIMAL MODELS OF AMYGDALA FUNCTIONAL AND ANATOMICAL CHANGES IN ASD

Due to the significant involvement of the amygdala and other limbic structures in social interactions, these brain areas have emerged as key focuses for ASD research (Seguin et al. 2021).

Amygdala dysfunction and structural alterations in early life are linked to autistic behaviours (Bachevalier 1994). Kemper and Bauman report that autistic children have enlarged amygdala with decreased neuron size in this region of the brain (Kemper and Bauman 1998). According to Schumann et al., autistic subjects have been found to exhibit structural alterations in the amygdala, including a decrease in neuron density and changes in volume (Schumann et al. 2004; Schumann and Amaral 2006). In particular, the size of the amygdala changes with age (Schumann et al. 2004). More recent studies have demonstrated a significant reduction in the number of neurons within the lateral nucleus of the amygdala in individuals with autism (Varghese et al. 2017). According to a study conducted by Seguin et al. (2021), adolescents with ASD exhibit enlarged basolateral amygdala nuclei when compared to their typically developing counterparts. Increased volumes of basolateral amygdala and cortical nuclei correlate positively with social behaviour deficits. The basolateral amygdala, a key brain region in emotion and motivational processing, relies on inhibitory GABAergic neurons for regulation. Disrupted GABAergic inhibition can result from the loss of GABAergic interneurons, altered GABA receptor function, or modulatory dysfunction. Disruptions in GABAergic control of the basolateral amygdala occur during development, aging, or after trauma, leading to hyperexcitability. This manifests as increased anxiety, emotional dysregulation, or seizures - behavioural alterations associated with ASD (Prager et al. 2016).

Conversely, larger volumes of the medial nuclei are negatively correlated with both social and communication deficits. Additionally, larger volumes of central amygdala nuclei are associated with heightened repetitive behaviours (Seguin et al. 2021).

Animal studies confirm that GABAergic interneurons in the basolateral amygdala are crucial for ASD-related behaviour (Prager et al. 2016). Rats are the most widely used animal models for evaluating the role of the amygdala in behaviours associated with autism. Rat amygdala damage models have shown behaviours such as stereotyped walking, impaired social play (Wolterink et al. 2001), and difficulties in social communication (Diergaarde et al. 2005). Furthermore, according to Paine et al. (2017), a reduction in GABA function within the basolateral amygdala is pathogenically linked to impaired social interaction.

Research using environmental ASD models and Fragile X knockout mice reveals impaired GABAergic signalling in the basolateral amygdala. This reduced inhibition arises from synaptic transmission deficits and disrupted GABA metabolism, but not from interneuron loss (Prager et al. 2016).

Notably, studies by Wang and colleagues on mice (Wang et al. 2018) revealed that nearly all neuronal nitric oxide synthase (nNOS)-positive cells in the basolateral amygdala are GABAergic inhibitory interneurons and that there was a reduction in nNOS-expressing cells in the basolateral amygdala of mouse models of autism (Wang et al. 2018). Thus, disruptions in the basolateral amygdala, caused by a reduction in nNOS interneurons and their synaptic connectivity, may contribute to the socio-emotional behavioural impairments observed in ASD (Wang et al. 2018).

Interestingly, studies on non-human primates show that the behavioural abnormalities in amygdala-lesioned animals align more closely with deficits in fear processing than with disruptions in social communication (Amaral et al. 2003). The findings of these authors suggest that infant rhesus monkeys raised by their mothers, even with selective bilateral lesions to the amygdala, exhibit a full range of species-typical social signals, including facial expressions, vocalisations, and body postures. Furthermore, throughout the first year of development, no signals of diminished emotional expression, motor stereotypes, or lack of social skills were observed (Amaral et al. 2003).

## ANIMAL MODELS OF ANATOMICAL ABNORMALITIES IN THE PREFRONTAL CORTEX IN ASD

The prefrontal cortex plays a crucial role in mammalian social behaviour, encompassing motivation, recognition, and decision-making. In humans, the medial prefrontal cortex plays a crucial role in complex social interactions, including self-awareness, understanding others’ perspectives, and emotion regulation. However, impaired prefrontal cortex function has been linked to several neuropsychiatric conditions, including ASD (Mohapatra and Wagner 2023).

A study conducted by Herbert and colleagues in 2004 investigated cerebral white matter volume in boys with autism, revealing an increase in the volume of “superficial” white matter, located directly beneath the cortex (Herbert et al. 2004). Additionally, Carper et al. (2002) reported that the frontal lobe exhibited the most significant magnitude of enlargement. In 2005, Carper and Courchesne found that a substantial portion of the frontal cortex undergoes enlargement during the early stage of autism. Notably, regions such as the orbital cortex and precentral gyrus show distinct variations in developmental anomalies, differing in magnitude or temporal progression (Carper and Courchesne 2005). Furthermore, early structural damage in the prefrontal cortex impairs social communication and cognition (Eslinger et al. 2004).

The prefrontal cortex evolved in stages. Agranular areas emerged first in early mammals and were shared among rodents and primates, including limbic regions like the agranular medial frontal cortex. Rodent studies provide insights into these shared areas, but less for primate-specific granular regions that dominate the human frontal lobe. These granular regions developed in early primates or their ancestors, including tree shrews, and further evolved in strepsirrhines and simians (apes, humans) (Preuss and Wise 2022).

Animal models provide evidence supporting the role of the human prefrontal cortex in social motivation (Mohapatra and Wagner 2023). According to Schneider and Koch (2005), damage to rats’ neonatal medial prefrontal cortex reduces social play, conditioned place preference linked to social contacts, and social grooming. In contrast, similar lesions in adult rats do not significantly affect social behaviour. Such findings suggest that prefrontal cortex anatomical abnormalities in ASD arise at an early stage of development.

Non-human primates (*Macaca fascicularis* and *Macaca mulatta*) are preferred models for ASD research due to their anatomical similarities with humans compared to rodents. Bussey et al. (2001) trained rhesus monkeys to establish new visuomotor connections during a single session. Following the bilateral removal of the orbital and ventral prefrontal cortex, the monkeys struggled to learn these associations within a single session that parallels aspects of ASD (Bussey et al. 2001). Similarly, Dias et al. (1996) noted in a letter to the Nature Journal that damage to the lateral prefrontal cortex in monkeys resulted in impaired inhibitory control over attentional selection. Conversely, damage to the orbitofrontal cortex led to a loss of control over affective processing, making it difficult for the monkeys to adjust their behaviour in response to changes in the emotional significance of stimuli (Dias et al. 1996). Ethical concerns dictate that research on monkeys should only occur under extraordinary conditions, thereby limiting the utilisation of monkey experimental models.

## CEREBELLAR ANATOMICAL ABNORMALITIES MODELS

Substantial evidence now suggests that the cerebellum is involved in a range of cognitive and emotional functions, including language, attention, fear, and pleasure responses (Mapelli et al. 2022). Cerebellar anatomical abnormalities are commonly observed in individuals with autism, highlighting the cerebellum’s role in the aetiopathogenesis of ASD (Bauman and Kemper 1985; Mapelli et al. 2022). A post-mortem human study by Bailey and colleagues found that ASD is associated with reduced size and number of Purkinje cells (Bailey et al. 1998). This finding aligns with data reported by Fatemi and colleagues, who noted that approximately 25% of individuals with ASD exhibit a reduction in Purkinje cell size (Fatemi et al. 2002). Moreover, growing evidence suggests that most individuals with ASD also exhibit motor impairments. In their original study, Freitag et al. (2007) observed that gross and fine motor impairments are associated with the severity of autistic symptoms, suggesting potential common pathogenetic mechanisms. In their comprehensive review, Fatemi et al. (2012) highlighted various cerebellar abnormalities in autistic patients, including structural alterations, inflammation, oxidative stress, altered neurotransmitter and protein levels, as well as motor and cognitive impairments related to the cerebellum.

In animal models for ASD with cerebellar abnormalities, mice are the most commonly used models for studying ASD due to their genetic manipulability, 85% similarity to human protein-coding genes, rapid reproduction cycle, and cost-effectiveness. Validated assays are employed to evaluate ASD-like phenotypes, and research on gene-targeted mouse models has enhanced our understanding of the pathogenic mechanisms underlying ASD. Monogenic models with cerebellar alterations can be categorised into syndromic, non-syndromic, and abnormal cerebellar development models (Mapelli et al. 2022).

Several genes crucial for the normal development of the cerebellum have been pathogenically linked to ASD. Notable examples include the engrailed homeobox 2 (*EN2*) gene, which plays a specific role in regulating the development of the mesencephalon and cerebellum, and the phosphatase and tensin homolog (PTEN) gene, an oncogene-suppressor gene involved in cell cycle control, apoptosis, and migration signalling (Mapelli et al. 2022). Interestingly, both *EN2* knockout mice (*EN2-KO*) and individuals with ASD display striking similarities in cerebellar morphological alterations, such as disrupted foliation patterning, hypoplasia, and a decreased number of Purkinje cells (Millen et al. 1994; Kuemerle et al. 1997). *EN2-KO* mice exhibit behaviours reminiscent of ASD, characterised by reduced sociability, impaired spatial memory, and heightened susceptibility to seizures (Provenzano et al. 2014). With specific deletion of *PTEN* in Purkinje cells (Purkinje cell *PTEN-KO)*, these neurons have altered morphology and reduced number (Cupolillo et al. 2016). Moreover, loss of *PTEN* in Purkinje cells leads to the development of ASD-like traits in mice, including impaired social communication, repetitive behaviour, and increased susceptibility to seizures (Cupolillo et al. 2016).

Additionally, genes such as *SHANK1-3* have been recently implicated in the pathogenesis of non-syndromic ASD (Mapelli et al. 2022). Notably, autistic subjects show *SHANK3* disruptions, often with a co-deletion of the Islet-Brain-2 (*IB2*) gene (Giza et al. 2010; Mapelli et al. 2022). Multiple *SHANK3* mutant mouse models for ASD have been developed, each with some construct validity. However, only one model accurately replicates a human *SHANK3* mutation- a model with a mutation that leads to a truncated SHANK3 protein lacking the C-terminal region (*SHANK3-∆C*) (Mapelli et al. 2022). *SHANK3-∆C* mice display social and behavioural abnormalities, novelty avoidance, and cerebellar alterations, including impaired motor coordination and altered density and morphology of the Purkinje cells (Kouser et al. 2013; Duffney et al. 2015; Kloth et al. 2015). *IB2-KO* mice also exhibit altered Purkinje cell morphology and typical ASD-like features, such as impaired social communication, motor deficits, and reduced exploratory behaviour (Giza et al. 2010). For a more in-depth review of cerebellar abnormalities in mouse models for ASD, refer to the recent comprehensive review by Mapelli et al. (2022).

Examples of non-syndromic and abnormal cerebellar development mouse models for ASD are summarised in Table 2.

**Table 2:** Examples of anatomical cerebellar abnormalities in mouse models of ASD

Mouse model	Cerebellum abnormalities	ASD features modelled	Study
*EN2-KO*	foliation hypoplasia, decreased number of Purkinje cells	abnormal social behaviour, impaired spatial memory, susceptibility to seizures	Millen et al. (1994); Kuemerle et al. (1997); Provenzano et al. (2014)
Purkinje cell *PTEN-KO*	altered morphology and decreased number of Purkinje cells	impaired social communication, repetitive behaviour, susceptibility to seizures	Cupolillo et al. (2016)
*SHANK3-∆C*	decreased Purkinje cell number with fewer dendritic spines	impaired social communication, repetitive behaviour, novelty avoidance	Kouser et al. (2013); Kloth et al. (2015); Duffney et al. (2015)
*IB2-KO*	altered Purkinje cell morphology: thinner Purkinje cell dendrites, shorter Purkinje cell dendritic arbour	impaired social communication, motor deficits, reduced exploratory behaviour	Giza et al. (2010)

ASD = autism spectrum disorder

In rodent ASD models, early cerebellar lesions cause visuomotor defects (Joyal et al. 1996), increase spontaneous motor activity, and reduce anxiety-like behaviour (Bobee et al. 2000).

## ENVIRONMENT-INDUCED ANIMAL MODELS FOR ASD

Environmental factors, whether alone or in combination with genetic influences, play a significant role in the development of ASD (Cheroni et al. 2020).

Experimental animal models that mimic environment-induced factors associated with ASD provide valuable insights into the pathogenic mechanisms underlying the disorder. These models are simple, quick to develop, and cost-effective (Li et al. 2021). Nonetheless, each animal model captures only a limited number of aspects related to the potential pathogenic mechanisms of ASD (Li et al. 2021). Environmental factors in ASD include maternal infection and prenatal exposure to chemicals such as valproic acid (Kim et al. 2016; Lavrov and Shabanov 2018), polychlorinated biphenyls (Jolous-Jamshidi et al. 2010), insecticides, of which the most common is chlorpyrifos (Lan et al. 2017), propionic acid, bisphenol propane and sevoflurane (Li et al. 2021), etc. Hence, environmental-induced animal models of ASD can be divided into chemical animal models (i.e., drug-induced models or chemical models) and non-chemical animal models (i.e., immune system modulation models, maternal immune activation models, or models involving other biological manipulations) (Ergaz et al. 2016; Ornoy et al. 2024).

## CHEMICAL ANIMAL MODELS FOR ASD – EXPOSURE TO VALPROIC ACID

Several studies have demonstrated that administering valproic acid during gestation for epilepsy treatment can induce symptoms resembling autism (Kim et al. 2016). Animal models using valproate have successfully reproduced ASD-like behaviours, offering a platform to investigate the neurobiological changes associated with this environmental factor (Patterson 2011).

The valproic acid model was created by Rodier et al. in 1996 (Rodier et al. 1996).

In line with symptoms seen in humans with autism, rodents (rats and mice) and zebrafish exposed prenatally to valproic acid exhibit increased repetitive and stereotypical behaviours (Schneider et al. 2008; Chen et al. 2018), communication deficits, and reduced interest in social novelty (Kim et al. 2016; Chen et al. 2018; Hirsch et al. 2020; Messina et al. 2024).

Hirsh and colleagues, using a rat model of autism induced by valproic acid exposure, reported typical ASD-like features in animals, such as impaired social communication, heightened anxiety, and lower sensitivity to pain (Hirsch et al. 2020). Additionally, Schneider et al. (2008) previously described gender differences in a rat model of autism induced by prenatal exposure to valproic acid: male offspring displayed a plethora of ASD-like features, including lower sensitivity to pain, repetitive/stereotypic-like behaviour, increased anxiety, and impaired social communication, whereas female pups exhibited mainly increased repetitive/stereotypic-like behaviour. While the precise mechanism by which prenatal exposure to valproic acid induces autism-like behaviours in both humans and rodents remains unclear, the rat model has been extensively validated and shows significant parallels with the behavioural, cellular, and molecular changes seen in individuals with autism (Nicolini and Fahnestock 2018).

In a mouse model for ASD, Ornoy et al. (2019) reported that pups treated with valproic acid also displayed neurobehavioural deficits, with more pronounced effects in males. In particular, male pups have shown impaired social communication and enhanced grooming activity, while female pups exhibited heightened anxiety.

Studies on zebrafish have shown that exposure to valproic acid leads to ASD-like phenotypes, including macrocephaly, hyperreactive movement, and altered social behaviours (Chen et al. 2018; Messina et al. 2024). Examples of valproic acid-induced ASD-like behavioural animal models are summarised in Table 3.

**Table 3:** Examples of valproic acid-induced ASD-like behavioural animal models

Animal	Valproic acid dosing regimen	Behavioural ASD features modelled	Study
Pregnant Wistar rats	intraperitoneal injection of 600 mg/kg on E12.5	male offspring: lower sensitivity to pain, repetitive/stereotypic-like behaviour, increased anxiety, impaired social communication female offspring: increased repetitive/stereotypic-like activity	Schneider et al. (2008)
Pregnant Wistar rats	intraperitoneal injection of 600 mg/kg on E12.5	lower sensitivity to pain, impaired social communication, increased anxiety	Hirsch et al. (2020)
ICR albino mice	subcutaneous injection of 300 mg/kg on PND4	male pups: impaired social communication, enhanced grooming activity female pups: increased anxiety	Ornoy et al. (2019)
Zebrafish embryos	exposure to concentrations of 5, 50, 500, 1 000, and 1 500 μM from 8 to 120 hpf	deficient social behaviour, hyperactive movement behaviour	Chen et al. (2018)
Zebrafish embryos	48 h exposure to 1 μM starting from 8 hpf	deficient social behaviour impaired social visual laterality	Messina et al. (2024)

ASD = autism spectrum disorder; E = embryonic day; hpf = hours post fertilisation; ICR = Institute of Cancer Research; PND = postnatal day

## NON-CHEMICAL ANIMAL MODELS FOR ASD – MATERNAL INFECTION/MATERNAL IMMUNE ACTIVATION

Studies have shown that maternal infections during pregnancy can increase the risk of neurodevelopmental disorders such as ASD in offspring. Activation of the maternal immune system by the inflammation process is considered a potential risk factor for abnormal brain development, potentially resulting in ASD development (Patterson 2011; Ornoy et al. 2024). Although the microbial pathogens or immune activators encountered in humans may vary from those studied in animal models, the autism-like effects observed in these models offer valuable insights into the underlying pathophysiology of ASD in humans.

Animal models replicating maternal infections have demonstrated behavioural abnormalities similar to those observed in individuals with ASD, highlighting the impact of prenatal environmental factors on neurodevelopment (Patterson 2011). Rodents, particularly mice and rats, are commonly used in experimental studies to investigate the effects of environmental factors on ASD-like behaviours (Hrabovska and Salyha 2016). Interestingly, maternal immune activation during pregnancy in rodents is associated with a dysregulated immune system in the offspring and also leads to autism-related phenotypes that persist into adulthood (Patterson 2011; Bruce et al. 2023).

The most commonly used animal models for maternal infection in ASD research include the prenatal Borna Disease Virus (BDV) infection model (Kim et al. 2016), prenatal lipopolysaccharide exposure model (Ornoy et al. 2024), and polyinosinic-polycytidylic acid (PolyIC) injection model (Kim et al. 2016; Ornoy et al. 2024). Indeed, rats exposed to prenatal BDV infection display stereotyped behaviour, reduced engagement in social play, and impaired social interactions, indicating distinct ASD-related phenotypes (Kim et al. 2016). Mice prenatally exposed to lipopolysaccharide exhibit more significant levels of anxiety (Wang et al. 2010) and social communication deficits (Kim et al. 2016). Similarly, offspring of rats injected with PolyIC, a double-stranded RNA that simulates maternal infection, display autism-related phenotypes (Kim et al. 2016).

Additionally, Bauman and colleagues reported interesting results from a non-human primate model (rhesus monkey) administered maternal immunoglobulin G (IgG) class antibodies purified from mothers of ASD children. An IgG-ASD offspring exhibited typical ASD-like behaviour, such as inappropriate socialisation, which deviated from species-typical social norms (Bauman et al. 2013).

Furthermore, genetic imbalances in synaptic connectivity may make individuals more susceptible to environmental disruptions during neurodevelopment, leading to altered neural networks and autism-related behaviours. Research supports this hypothesis by linking changes in synaptic connectivity to autism-like behaviours. For example, mutations in genes responsible for metabolising xenobiotics have been associated with a higher risk of ASD. Impaired detoxification of environmental chemicals can amplify the neurotoxic effects of environmental pollutants (Bjorklund et al. 2021; Keil-Stietz and Lein 2023). According to Balaguer-Trias and colleagues, the gut microbiota is crucial in neurodevelopmental processes (Balaguer-Trias et al. 2022). In autistic children, distinct differences have been observed in their gut microbiota compared to neurotypical children.

Additionally, toxicological studies highlight a reciprocal relationship: the gut microbiota influences how xenobiotics are metabolised, and conversely, exposure to environmental chemicals can alter the balance of the gut microbiota (Balaguer-Trias et al. 2022; Keil-Stietz and Lein 2023). However, it remains unclear how genetic and environmental factors influence the risk of autism (Keil-Stietz and Lein 2023).

Research on mice conducted by Hsiao and colleagues has demonstrated that the gut microbiota can influence animal behaviour by modulating neuroactive metabolites. This suggests a strong connection between the gut-brain axis and the development of the underlying pathophysiological mechanisms of ASD (Hsiao et al. 2013). Furthermore, after transplanting gut microbiota from human donors with ASD into germ-free mice, the results indicate that colonisation with ASD-associated microbiota is sufficient to elicit core autistic behaviours (Sharon et al. 2019). These studies suggest that behavioural abnormalities may arise from host genetics and microbial influences, prompting a re-evaluation of neurological diseases.

## ASSESSMENT OF CURRENT ANIMAL MODELS AND THE DEVELOPMENT OF NOVEL ANIMAL MODELS FOR ASD

The effectiveness of an experimental animal model is directly proportional to its ability to replicate human diseases accurately. At present, nearly all existing animal models for ASD replicate typical autistic features, as they exhibit common clinical symptoms such as stereotyped behaviours and impaired social interactions. However, except for songbirds, none of these experimental models effectively addresses the linguistic deficits observed in autistic patients (Li et al. 2021). While an ideal therapeutic approach for addressing impaired social communication and repetitive behaviours in autistic subjects has yet to be established, existing ASD animal models demonstrate a certain degree of predictive validity in evaluating treatment effectiveness (Li et al. 2021).

Moreover, due to the significant heterogeneity of ASD, there is no consensus on the most suitable animal model for studying its pathophysiological aspects. Identifying common underlying causes across independent models presents a valuable opportunity to uncover novel factors pathogenically linked to ASD.

Alongside the use of current ASD models, it is essential to create new models that incorporate species with a closer evolutionary relationship to humans. The tree shrew (*Tupaia belangeri*) has been recognised as a promising alternative to non-human primates in research due to its close evolutionary ties to primates. Advancements, such as genome sequencing, genetic manipulation, and brain atlas creation, have enhanced experimental research capabilities (Yao et al. 2024). Tree shrews possess a more developed nervous system and stress response that closely resembles those of humans, indicating their great potential as effective experimental models for studying diseases with behavioural abnormalities. They outperform rodents in cognitive tasks such as reverse and reward-punishment anticipation (Ohl and Fuchs 1999). Tree shrews exhibit strong novelty preferences akin to those observed in both rodents and primates (Khani and Rainer 2012). Ni and colleagues found that male tree shrews exhibited social avoidance behaviour, while male mice displayed prosocial behaviour toward unfamiliar conspecifics. This suggests that tree shrews could serve as a novel animal model, distinct from mice, for investigating alterations in social behaviours (Ni et al. 2020). Tree shrews are employed in models of social frustration, learned helplessness, and chronic mild stress models (Meng et al. 2016), positioning them as key models for studying ASD pathophysiology. However, the global scarcity of these animals presents a significant challenge, along with the difficulty of identifying key research questions best studied for this species (Yao et al. 2024).

Pigs (*Sus scrofa domesticus*) have also become especially significant in modelling human diseases, particularly neurological disorders, due to their anatomical and physiological similarities to humans. Notably, their brain structure and function closely resemble those of humans, making them a promising model for investigating neurological conditions in humans (Li et al. 2021; Yuan et al. 2024). In their recent study, Yuan and colleagues explored using a miniature Bama pig model to study ASD by exposing embryos to valproic acid. These results showed behavioural changes like abnormal gait, anxiety, impaired learning, and altered social behaviour, along with significant neuroanatomical changes similar to those in ASD (Yuan et al. 2024). These results position pigs as a transitional bridge between rodent studies and primate-based research, offering an ethical, clinically relevant model for probing neurodevelopmental disorders such as ASD.

## CONCLUSIONS

Suitable animal models of human diseases are crucial for understanding their aetiopathogenic aspects. While diseases with biological markers have well-defined models, non-genetic models for neurobehavioural and neuropsychiatric disorders often lack these markers. As a result, autistic-like behaviours in animals are challenging to define, as specific neurobehavioural traits may not accurately reflect human behaviour.

Until now, each animal model has primarily focused on a single genetic, neuronal, behavioural, or other pattern rather than employing a comprehensive approach. The field has reached a point where combining and further exploration of animal models are needed. Only a complex model can enhance our understanding of the interactions between the physiological and behavioural features of ASD. Most animal models used in autism research involve rodents, which are often considered the most convenient species for this purpose. However, behavioural results in mice can sometimes conflict with human symptoms. These discrepancies can largely be attributed to the laboratory environment and the genetic background of the transgenic rodents. Mammalian species, particularly non-human primates, offer the best opportunity to address specific pathophysiological hypotheses due to their relatively high anatomical and phylogenetic similarity to humans. Nonetheless, ethical considerations necessitate that experimental studies on non-human primates be strictly limited to exceptional circumstances. Currently, tree shrew models serve as an alternative to non-human primate models; however, their limited global availability poses a significant challenge in the field of research. Hence, when conducting experimental studies on animals, each modality should be examined separately to understand the effects of each mechanism. Subsequently, more complex models should be developed that integrate all these properties.
